# Combined serum neurofilament light chain and Tau as biomarkers of diabetic peripheral neuropathy

**DOI:** 10.3389/fendo.2026.1705881

**Published:** 2026-01-21

**Authors:** Ya Chen, Shanshan Huang, Wenting Cai, Jinfang Song, Chenyan Sui

**Affiliations:** 1 Department of Endocrinology, Affiliated Hospital of Jiangnan University, Jiangnan University, Wuxi, China; 2 Department of Pharmacy, Affiliated Hospital of Jiangnan University, Jiangnan University, Wuxi, China; 3 Department of Neurology, Affiliated Hospital of Jiangnan University, Jiangnan University, Wuxi, China

**Keywords:** combined biomarkers, diabetic peripheral neuropathy, neurofilament light chain (NFL), screening performance, Tau protein

## Abstract

**Background:**

To assess the diagnostic performance of combined serum neurofilament light chain (NfL) and Tau protein measurement in detecting diabetic peripheral neuropathy (DPN).

**Methods:**

We retrospectively analyzed 50 patients with DPN and 50 age- and sex-matched Non-DPN treated between December 2023 and May 2024. Serum NfL and Tau were measured by ELISA assays. Logistic regression identified factors associated with DPN, and receiver operating characteristic (ROC) curves assessed diagnostic performance.

**Results:**

Compared with controls, DPN patients had longer diabetes duration, higher blood pressure, BMI, and HbA1c, and lower motor and sensory nerve conduction velocities (all P < 0.05). Serum NfL and Tau were significantly elevated in the DPN group (P < 0.001). Multivariate logistic analysis showed diabetes duration, systolic blood pressure, and elevated NfL and Tau were independently associated with DPN. ROC analysis revealed AUCs of 0.825 for Tau, 0.829 for NfL, and 0.896 for their combination, with combined detection achieving sensitivity of 93.9% and specificity of 82.4%.

**Conclusions:**

Elevated serum NfL and Tau levels are independently associated with DPN. The combined assessment may provide a complementary, non-invasive approach for preliminary screening and risk stratification of diabetic peripheral neuropathy.

## 1 Introduction

Diabetes has emerged as one of the most serious global health challenges of the 21st century. According to the 10th edition of the International Diabetes Federation (IDF) Diabetes Atlas, an estimated 537 million adults worldwide were living with diabetes in 2021, and this number is projected to rise to 643 million by 2030 and 783 million by 2045 (1). Moreover, more than 6.7 million deaths annually are attributed to diabetes-related causes, and global health expenditures for diabetes are approaching one trillion USD, with a substantial proportion of cases remaining undiagnosed (2). These statistics underscore the urgent need for early detection and effective management strategies to mitigate disease progression and its complications.

Among diabetes-related complications, diabetic peripheral neuropathy (DPN) is one of the most common and debilitating, affecting nearly half of patients with diabetes (3, 4). Epidemiological data indicated that the risk of DPN increases progressively with both diabetes duration and age. Diabetic sensorimotor polyneuropathy may already be present in approximately 10%–15% of patients at the time of type 2 diabetes diagnosis, and the prevalence has been reported to rise to around 50% after ~10 years of disease duration, underscoring that a substantial burden of neuropathy develops relatively early in the disease course (4). DPN frequently develops insidiously, with many individuals remaining asymptomatic until advanced stages, when irreversible nerve damage has already occurred (5). In severe cases, DPN can lead to chronic pain (6), foot ulcers (7), amputations (8), and even premature mortality (8, 9). Despite its high prevalence and serious outcomes, no disease-modifying treatment is currently available. Current diagnostic methods rely largely on clinical symptoms, neurological examination, and nerve conduction velocity (NCV) testing—the latter regarded as the gold standard but limited by operator dependence, high cost, and unsuitability for large-scale screening (10). Therefore, there is an urgent clinical need for reliable, minimally invasive biomarkers to facilitate early identification of DPN.

Neurofilament light chain (NfL), a key structural protein of large myelinated axons, has been established as a sensitive marker of axonal injury in a variety of central nervous system disorders (11). Elevated circulating NfL levels have also been reported in patients with DPN, suggesting its potential diagnostic utility (12, 13). Tau, a microtubule-associated protein primarily investigated in Alzheimer’s disease and other neurodegenerative disorders (14, 15), is also expressed in peripheral nerves and may play a role in axonal stability and repair (16). However, its role in DPN has not been systematically explored.

Both NfL and Tau are established indicators of neuronal injury, reflecting axonal damage (17) and microtubule dysfunction (18), respectively. While circulating NfL has been increasingly explored as a marker of axonal injury in DPN (12), the clinical relevance of serum Tau in DPN remains largely unexplored. More importantly, whether the combined assessment of these two complementary indicators of neuroaxonal injury provides incremental diagnostic value beyond individual biomarkers has not been systematically evaluated. In this study, we performed a head-to-head evaluation of serum NfL and Tau in patients with and without DPN, and anchored their associations to objective electrophysiological impairment assessed by nerve conduction studies. We also quantitatively evaluated the added diagnostic value of a combined biomarker model. By directly comparing individual and combined biomarker performance, our work provides novel evidence supporting the complementary utility of NfL and Tau as a non-invasive screening approach for DPN.

## 2 Materials and methods

### 2.1 Study design and subjects

This was a cross-sectional case-control study conducted at the Department of Endocrinology, Affiliated Hospital of Jiangnan University. A total of 100 participants were included between December 2023 and May 2024, comprising 50 patients with DPN and 50 age- and sex-matched patients with non-DPN (1:1 matching). All patients had complete clinical records and follow-up data, and provided written informed consent. The diagnosis of type 2 diabetes mellitus was established according to the diagnostic criteria of the American Diabetes Association (ADA), defined by one or more of the following: fasting plasma glucose ≥7.0 mmol/L (126 mg/dL), 2-hour plasma glucose ≥11.1 mmol/L (200 mg/dL) during an oral glucose tolerance test, HbA1c ≥6.5%, or a prior clinical diagnosis documented in the medical record with ongoing antidiabetic treatment (4). Electrophysiological confirmation of DPN was obtained using standardized nerve conduction studies of the median nerve. Motor nerve conduction velocity (MNCV), sensory nerve conduction velocity (SNCV), distal motor latency, and sensory nerve action potential amplitude were assessed. Based on established reference values for adult populations, abnormal nerve conduction was defined as motor nerve conduction velocity <50–55 m/s and/or sensory nerve conduction velocity <45–50 m/s, or prolonged distal motor latency, after age adjustment (19, 20). Final determination of abnormality was made when any electrophysiological parameter fell below the lower limit of normal for age-matched reference ranges provided by the electrodiagnostic laboratory. A diagnosis of DPN required the presence of at least one abnormal electrophysiological parameter in combination with compatible clinical symptoms or signs (19). Inclusion criteria for the DPN group were as follows: (1) a clinically confirmed diagnosis of type 2 diabetes mellitus according to established diagnostic criteria; (2) a diagnosis of diabetic peripheral neuropathy based on the presence of typical clinical symptoms or signs of peripheral neuropathy (such as numbness, paresthesia, pain, or reduced tendon reflexes) in combination with objective electrophysiological evidence of impaired nerve conduction velocity. Inclusion criteria for the Non-DPN group were as follows: (1) a clinically confirmed diagnosis of type 2 diabetes mellitus; (2) absence of clinical symptoms or signs of peripheral neuropathy; (3) normal findings on nerve conduction studies. Exclusion criteria for the both groups: (1) peripheral neuropathy due to other causes (e.g. alcohol abuse, vitamin deficiencies, toxic exposure, infections, or hereditary neuropathies); (2) severe dysfunction of major organs (e.g. hepatic, renal, or cardiac failure); (3) malignancies under active treatment; (4) autoimmune or other systemic immune-related disorders; (5) central nervous system diseases (e.g. stroke, Parkinson’s disease, multiple sclerosis, or demyelinating disorders); (6) a history of limb trauma or surgery that could affect nerve function; (7) use of neurotoxic drugs within the past 6 months.

### 2.2 Clinical data collection

Demographic and clinical characteristics were collected for all participants at the time of enrollment. The recorded variables included sex, chronological age, body mass index (BMI), systolic and diastolic blood pressure, and the duration of diabetes since initial diagnosis. These data were obtained from standardized medical records and routine clinical assessments to ensure accuracy and consistency across the study population.

### 2.3 Specimen collection and laboratory analyses

Fasting venous blood samples (5 mL) were collected from all participants and centrifuged at 3500 rpm for 10 minutes to obtain serum. Biochemical parameters were measured as follows: fasting plasma glucose was determined using the glucose oxidase method (Roche, Germany); HbA1c was assessed by the cyanmethemoglobin method (Bio-Rad, USA); serum insulin and C-peptide levels were quantified by chemiluminescence immunoassays (Abbott, USA); and total cholesterol and triglycerides were analyzed using enzymatic colorimetric methods (Beckman Coulter, USA). Serum NfL and Tau protein concentrations were measured using commercially available enzyme-linked immunosorbent assay (ELISA) kits (R&D, USA) according to the manufacturer’s instructions.

### 2.4 Neurophysiological examination

MNCV and SNCV of the median nerves were assessed in all participants using a Viking IV electrodiagnostic system (Nicolet, USA). Examinations were performed under standardized conditions by experienced neurophysiologists. Skin temperature was maintained at 32–34 °C. Standard stimulation parameters were used, including supramaximal stimulation intensity (20–40 mA) and filter settings of 20 Hz–10 kHz. The procedures followed established guidelines for peripheral nerve conduction studies.

### 2.5 Statistical analysis

Continuous variables with a normal distribution were expressed as mean ± standard deviation (SD), while non-normally distributed continuous variables were presented as median and interquartile range (IQR). Categorical variables were presented as frequencies and percentages. Normality was assessed using the Kolmogorov–Smirnov test. For between-group comparisons, independent-samples t tests were applied to normally distributed variables, while Mann–Whitney U tests were used for non-normally distributed variables. Categorical data were analyzed using the χ² test.

Correlations between serum biomarker levels (NfL, Tau) and nerve conduction velocities (MNCV, SNCV) were examined using Spearman’s correlation analysis with linear regression. In logistic regression analyses, DPN status was coded as the dependent variable (DPN = 1, non-DPN = 0) and sex was coded as a binary variable (female = 0, male = 1), variables with univariate *P* < 0.10 were included in multivariate models. To ensure comparability and reduce potential problems due to scale differences, we standardized the two biomarkers, NfL and Tau protein. We converted these variables into z-scores with a mean of 0 and a standard deviation of 1. The combined NfL+Tau index was generated using a multivariable logistic regression model, with NfL and Tau entered simultaneously as continuous predictors. The predicted probability from this model was used for ROC analysis. ROC curve analysis was conducted to evaluate the diagnostic performance of serum NfL, Tau, and their combination. Optimal cut-off values were determined by maximizing Youden’s index for each ROC curve. Sensitivity, specificity, and the area under the curve (AUC) were calculated accordingly. For multiple comparisons, Bonferroni correction was applied. A two-tailed *P* < 0.05 was considered statistically significant. Analyses were performed using SAS version 9.4 (SAS Institute, USA).

## 3 Results

### 3.1 Comparison of clinical characteristics

The demographic and clinical characteristics of the study population are shown in **Table 1**. No significant differences were observed between the DPN and non-DPN groups with respect to age (median 55 *vs*. 58 years, *P* = 0.103) or sex distribution (66.0% *vs*. 58.0% male, *P* = 0.412). However, patients in the DPN group exhibited significantly higher BMI (25.92 ± 2.83 *vs*. 23.85 ± 3.40 kg/m², *P* < 0.001) and longer diabetes duration (3.00 [0.94–8.00] *vs*. 1.00 [0.62–1.50] years, *P* < 0.001) compared with those in the non-DPN group. Regarding blood pressure, DBP was markedly elevated in the DPN group (85 (77–93) *vs*. 70 (65–75) mmHg, *P* < 0.001), whereas SBP did not differ significantly between groups. Glycemic and metabolic parameters showed that the DPN group had substantially higher HbA1c levels (8.60 [7.60–10.57] *vs*. 6.65 [6.10–7.60] %, *P* < 0.001) and lower C-peptide concentrations (1.59 [0.96–2.22] *vs*. 1.89 [1.46–2.33] pg/mL, *P* = 0.041). No significant differences were observed in fasting blood glucose, insulin, triglyceride, or total cholesterol levels (*P* > 0.05 for all).

**Table 1:** Demographic and clinical characteristics of study participants.

Parameters	Overall (n=100)	DPN group (n = 50)	Non-DPN group (n = 50)	*P*-value
Age (years) (median, IQR)	56.5 (45.5, 65)	55 (45, 60)	58 (49, 67)	0.103
Gender (n, %)				0.412
Male	62 (62.00%)	33 (66.00%)	29 (58.00%)	
Female	38 (38.00%)	17 (34.00%)	21 (42.00%)	
BMI (kg/m^2^) (median, IQR)	24.89 ± 3.29	25.92 ± 2.83	23.85 ± 3.40	<0.001
DM duration (years)	1.00(0.73,3.25)	3.00 (0.94, 8.00)	1.00 (0.62, 1.50)	<0.001
SBP (mmHg) (median, IQR)	132 (121, 140)	135 (121, 142)	130 (121, 139)	0.371
DBP (mmHg) (median, IQR)	75 (70, 86)	85 (77, 93)	70 (65, 75)	<0.001
FBG (mmol/L) (mean ± SD)	8.43 ± 3.32	7.62 ± 1.94	9.25 ± 4.14	0.074
HbA1c (%) (median, IQR)	7.60 (6.38, 9.90)	8.60 (7.60, 10.57)	6.65 (6.10, 7.60)	<0.001
Insulin (pmol/L) (median, IQR)	9 (7, 12)	10 (6, 14)	9 (7, 11)	0.612
C peptide (pg/mL) (median, IQR)	1.76 (1.32, 2.33)	1.59 (0.96, 2.22)	1.89 (1.46, 2.33)	0.041
Triglyceride (mmol/L) (median, IQR)	1.61 (1.06, 2.59)	1.75 (1.24, 2.63)	1.53 (1.00, 2.37)	0.191
Cholesterol (mmol/L) (mean ± SD)	4.80 ± 1.18	4.79 ± 1.15	4.81 ± 1.21	0.780

DM, diabetes mellitus; BMI, body mass index; SBP, systolic blood pressure; DBP, diastolic blood pressure; FBG, Fasting blood glucose; HbA1c, hemoglobin A1c

### 3.2 Serum NfL and Tau levels and their correlation with nerve conduction

**Figure 1:**
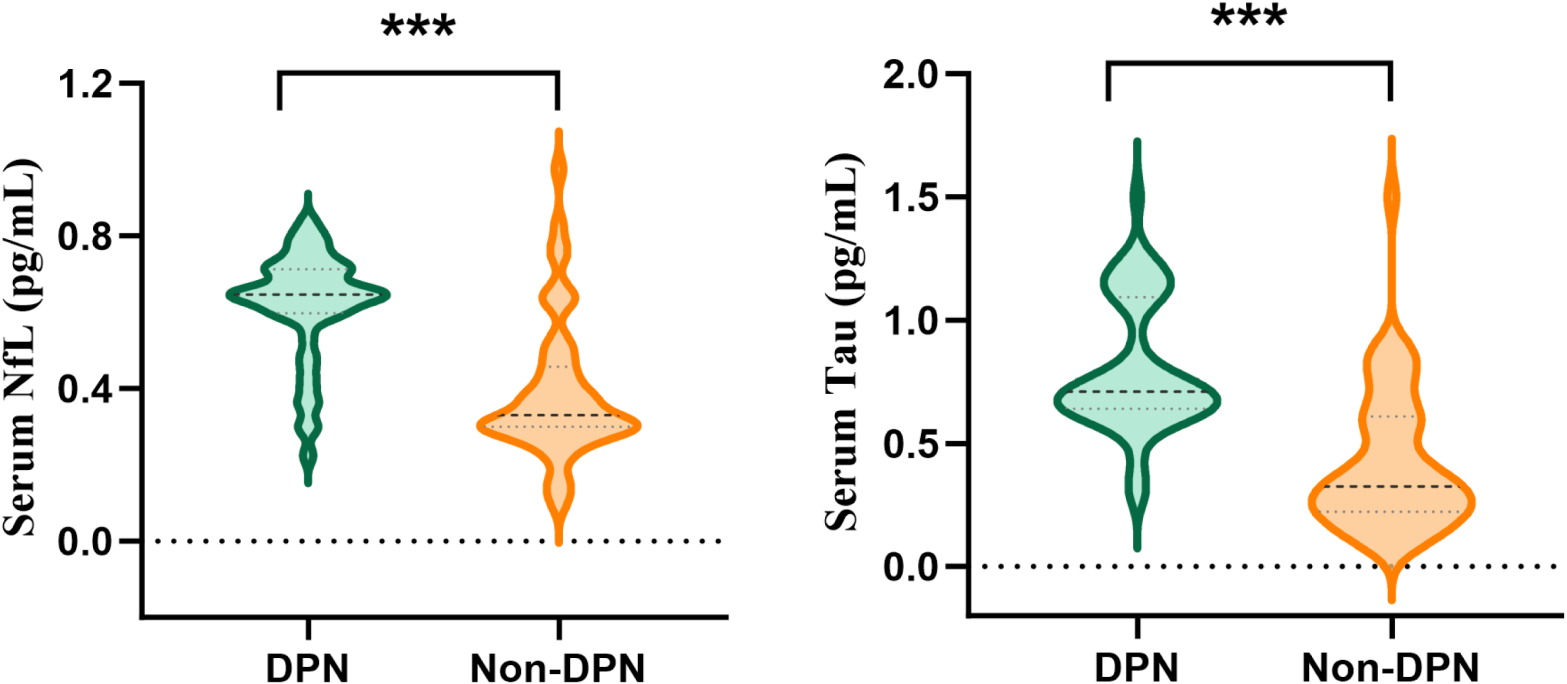
Serum NfL and Tau levels in DPN and non-DPN groups. Serum NfL and Tau levels were significantly higher in the DPN group compared with the non-DPN group (*P* < 0.001). Data are presented as the median with interquartile range (IQR), *** *p* < 0.001.

**Figure 2:**
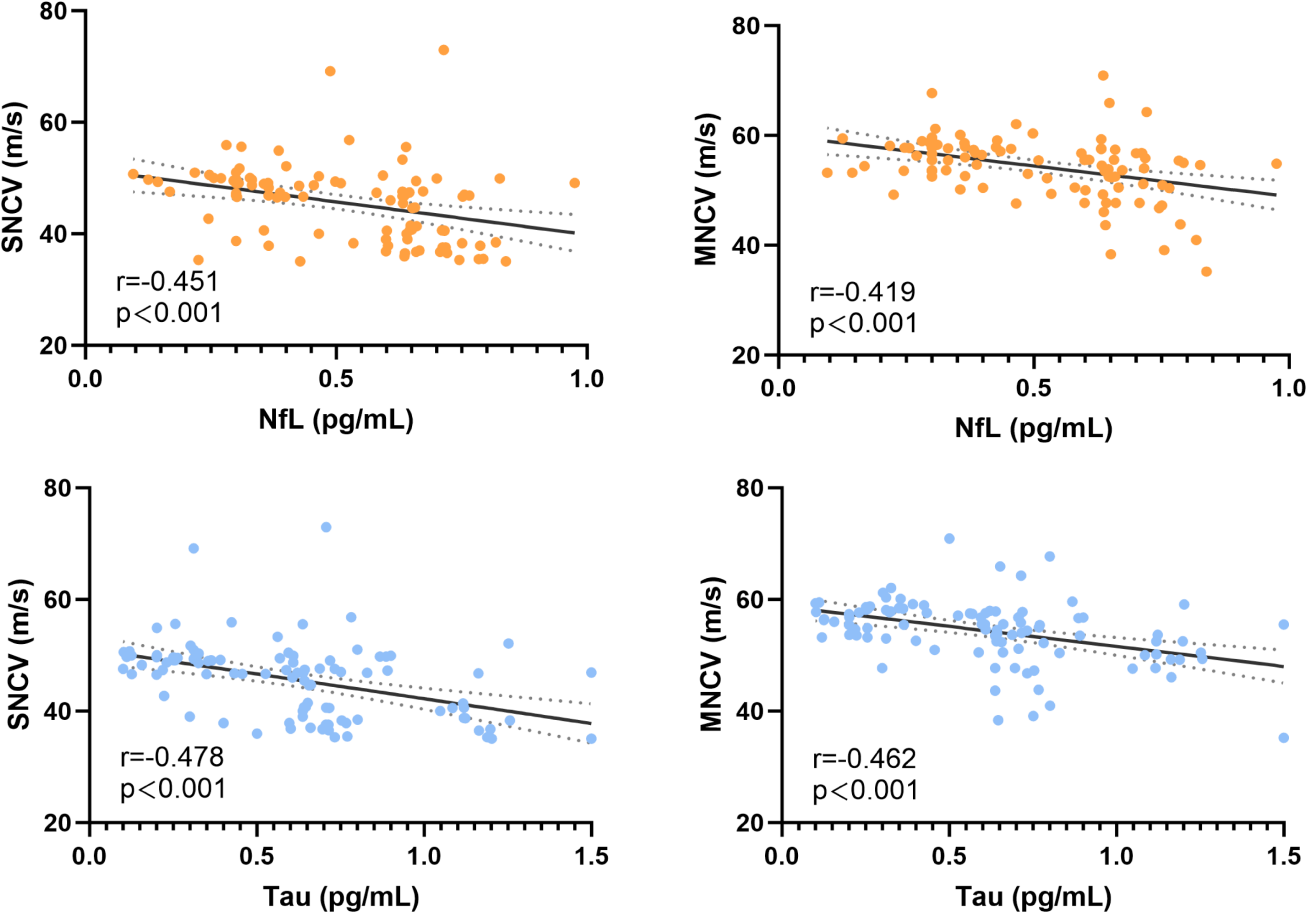
Correlations of serum NfL and Tau levels with nerve conduction velocities. Serum NfL and Tau levels were negatively correlated with motor nerve conduction velocity (MNCV) and sensory nerve conduction velocity (SNCV) in DPN patients. Correlations were assessed using Spearman’s rank correlation analysis. Solid lines represent fitted regression lines; dashed lines indicate 95% confidence intervals, *** *p* < 0.001.

Serum concentrations of NfL and Tau protein were markedly elevated in patients with DPN compared with those without neuropathy (**Figure 1**). Mean NfL levels were 0.62 ± 0.14 pg/mL in the DPN group versus 0.39 ± 0.18 pg/mL in the non-DPN group (*P* < 0.001). Similarly, Tau concentrations were significantly higher in the DPN group (0.80 ± 0.27 pg/mL) than in the non-DPN group (0.42 ± 0.28 pg/mL; *P* < 0.001). Correlation analyses further demonstrated that serum NfL levels were inversely associated with both motor nerve conduction velocity (MNCV; r = –0.419, *P* < 0.001) and sensory nerve conduction velocity (SNCV; r = –0.451, *P* < 0.001). Likewise, Tau concentrations were negatively correlated with MNCV (r = –0.462, *P* < 0.001) and SNCV (r = –0.478, *P* < 0.001) (**Figure 2**). These findings suggested that higher circulating NfL and Tau levels indicate a more severe impairment of peripheral nerve conduction in patients with DPN.

### 3.3 Logistic regression analysis of clinical and biomarker factors related to DPN

Logistic regression analyses were conducted to identify factors associated with the presence of DPN (**Table 2**). In univariate analysis, longer diabetes duration, higher HbA1c, elevated diastolic blood pressure, and increased serum NfL and Tau concentrations were all significantly related to DPN (P < 0.05 for all). In multivariate analysis, after adjusting for potential confounders, diabetes duration remained significantly associated with DPN (OR = 1.795, 95% CI: 1.057–3.049, *P* = 0.030). Importantly, both serum NfL (OR >999.999, 95% CI: 23.500->999.999), *P* = 0.002) and Tau (OR = 107.647, 95% CI: 6.050->999.999, *P* = 0.001) were independently associated with DPN status.

**Table 2:** Logistic regression analysis of factors associated with DPN.

Parameters	Univariate		Multivariate	
	OR (95%CI)	*P* value	OR (95%CI)	*P* value
Age (years)	0.977 (0.948-1.006)	0.113		
Gender	1.406 (0.625-3.163)	0.411		
BMI (kg/m^2^)	1.236 (1.077-1.418)	0.003	0.951 (0.723-1.251)	0.721
DM duration (years)	1.562 (1.222-1.998)	<0.001	1.795 (1.057-3.049)	0.030
SBP (mmHg)	1.013 (0.987-1.041)	0.327		
DBP (mmHg)	1.074 (1.034-1.116)	<0.001	1.073 (0.989-1.164)	0.090
FBG (mmol/L)	1.187 (1.027-1.371)	0.020	0.936 (0.696-1.261)	0.665
HbA1c (%)	1.611 (1.269-2.045)	<0.001	1.497 (0.945-2.370)	0.086
Insulin (pmol/L)	0.995 (0.984-1.006)	0.390		
C peptide (pg/mL)	0.751 (0.483-1.167)	0.203		
Triglyceride (mmol/L)	1.303 (0.995-1.706)	0.055	2.319 (1.046-5.144)	0.039
Cholesterol (mmol/L)	1.012 (0.724-1.415)	0.944		
NfL (pg/mL)	4.640 (2.600- 8.280)	<0.001	5.519 (1.871-16.280)	0.002
Tau (pg/mL)	5.728 (2.802-11.707)	<0.001	4.736 (1.819- 12.331)	0.001

DM, diabetes mellitus; BMI, body mass index; SBP, systolic blood pressure; DBP, diastolic blood pressure; FBG, Fasting blood glucose; HbA1c, hemoglobin A1c; NfL, Neurofilament light

Univariate: Univariate logistic regression analysis

Multivariate: Multivariate logistic regression analysis: inclusion of predictors exhibiting univariate logistic regression outcomes with *P*<0.10

Sex was coded as a binary variable (female = 0, male = 1), with female as the reference category.

### 3.4 Diagnostic value of serum NfL and Tau in DPN

**Figure 3:**
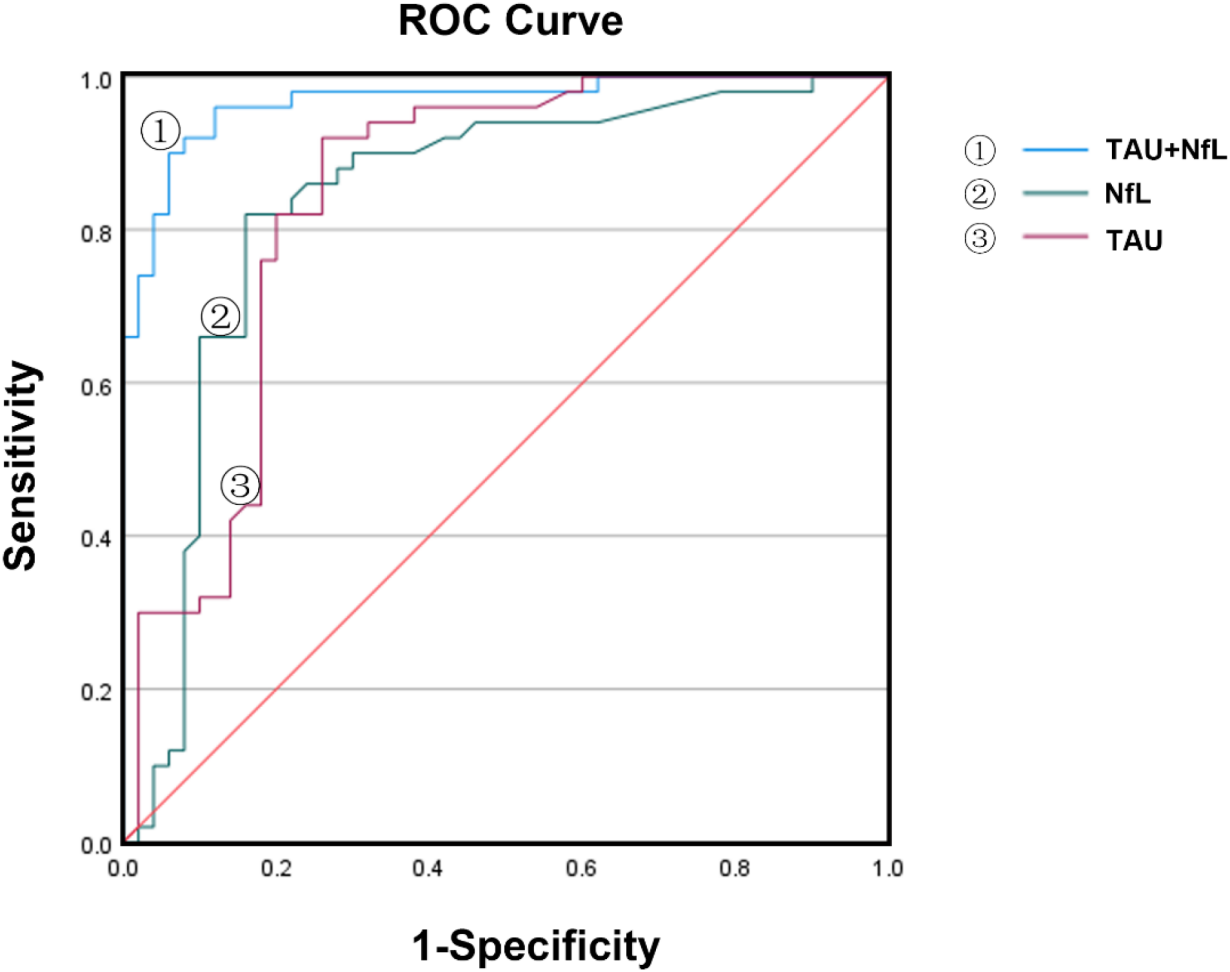
Diagnostic performance of serum NfL, Tau, and their combination for DPN. Receiver operating characteristic (ROC) curves demonstrate the diagnostic value of serum NfL, Tau, and their combined assessment in detecting DPN.

ROC curve analysis was conducted to assess the diagnostic utility of serum NfL and Tau (**Table 3**, **Figure 3**). The area under the curve (AUC) values for NfL and Tau alone were 0.829 (95% CI: 0.744–0.913) and 0.825 (95% CI: 0.738–0.913), respectively, indicating good diagnostic performance. When combined, NfL and Tau yielded an AUC of 0.896 (95% CI: 0.829–0.962), with sensitivity of 93.9% and specificity of 82.4%, markedly superior to either biomarker alone. These findings suggest that the combined assessment of serum NfL and Tau substantially enhances diagnostic accuracy for DPN compared with individual biomarker evaluation.

**Table 3:** Diagnostic performance of serum NfL, Tau, and their combination for DPN.

Index	AUC	95% CI LL	95% CI UL	Cut-off point	Sensitivity	Specificity	Youden’s index
Tau	0.825	0.738	0.913	0.529	0.816	0.824	0.64
NfL	0.829	0.744	0.913	0.466	0.918	0.725	0.643
NfL+Tau	0.896	0.829	0.962	0.507	0.939	0.824	0.763

NfL, Neurofilament light

## 4 Discussion

This study investigated the diagnostic value of serum NfL and Tau protein in patients with DPN. We found that serum concentrations of both biomarkers were significantly higher in DPN patients compared with Non-DPN patients. Elevated NfL and Tau levels were closely linked to peripheral nerve dysfunction, as demonstrated by their strong inverse correlations with motor and sensory nerve conduction velocities. Logistic regression further indicated that both proteins were independently associated with the presence of DPN. Importantly, the combined assessment of NfL and Tau provided superior diagnostic accuracy relative to either biomarker alone, underscoring their potential complementary value in the identification of DPN.

NfL is a structural protein abundantly expressed in large myelinated axons and is released into body fluids upon axonal injury (21). Previous studies have established NfL as a robust biomarker for central nervous system disorders such as multiple sclerosis (22), amyotrophic lateral sclerosis (23), and Alzheimer’s disease (24, 25). More recent investigations have extended its utility to peripheral neuropathies, with elevated serum NfL reported in DPN and related conditions (26). Our findings are consistent with these observations, confirming that NfL levels are significantly higher in DPN patients and are negatively correlated with both MNCV and SNCV. From a mechanistic perspective, chronic hyperglycemia, oxidative stress, and microvascular dysfunction in diabetes are known to promote progressive axonal degeneration, leading to the release of structural axonal proteins such as NfL into the circulation. Therefore, elevated serum NfL levels in DPN likely reflect ongoing peripheral axonal injury rather than nonspecific neuronal damage (27). This supports the concept that circulating NfL reflects the degree of peripheral axonal damage and may serve as a non-invasive indicator of nerve dysfunction in DPN.

Tau, a microtubule-associated protein, has been extensively studied in neurodegenerative diseases where abnormal phosphorylation leads to microtubule destabilization and axonal degeneration (28). Such pathological modifications impair axonal transport, disrupt synaptic function, and ultimately contribute to neuronal loss (29). Emerging evidence further indicates that circulating Tau levels may serve as a surrogate marker of ongoing neurodegeneration, linking molecular changes to measurable clinical outcomes (30). However, its role in peripheral nerve disorders remains poorly defined. Experimental studies have suggested that Tau participates in axonal growth and regeneration in the peripheral nervous system (31). Notably, increasing experimental evidence supports a role of Tau in peripheral nerve biology, including Schwann cell behavior, cytoskeletal organization, and axonal regeneration after injury (16, 32). These findings provide a plausible explanation that elevated serum Tau in DPN may reflect diabetes-related cytoskeletal disruption and impaired axonal maintenance in the peripheral nervous system. In our study, serum Tau concentrations were significantly elevated in DPN patients and showed strong negative correlations with MNCV and SNCV. Logistic regression further demonstrated that Tau was independently associated with DPN, suggesting that Tau may reflect microtubule dysfunction and contribute to the pathophysiology of diabetic neuropathy. To our knowledge, this is among the first studies to implicate Tau as a potential biomarker in DPN, highlighting a novel area for further exploration.

Patients with DPN in the present study exhibited a longer duration of diabetes and poorer glycemic control, as reflected by higher HbA1c levels, compared with those without neuropathy. Prolonged exposure to hyperglycemia and metabolic stress is known to drive cumulative neuroaxonal injury through mechanisms such as oxidative stress (33), microvascular dysfunction, and impaired axonal transport (34). In this context, elevated serum NfL and Tau levels may partly reflect the cumulative metabolic burden and disease duration associated with diabetes, rather than neuropathic injury alone. In addition, NfL and Tau are not specific to peripheral nerves and are well-established markers of neuroaxonal injury in the central nervous system. Diabetes is associated with accelerated brain atrophy (35), cerebral small-vessel disease (36), and cognitive decline (37), all of which may contribute to increased circulating levels of these biomarkers. Therefore, elevated serum NfL and Tau likely represent integrated signals of diabetes-related neuroaxonal damage arising from both central and peripheral nervous system involvement.

Although both NfL and Tau were individually associated with DPN, their diagnostic performance as single biomarkers was moderate (AUC≈0.83). When combined, however, diagnostic accuracy improved markedly (AUC 0.896, sensitivity 93.9%, specificity 82.4%). This improvement likely reflects the complementary biological information provided by these proteins: NfL primarily reflects axonal integrity (38), whereas Tau reflects microtubule stability (39). Thus, their combined evaluation may provide a more comprehensive picture of peripheral nerve injury in diabetes. Conventional diagnostic approaches for DPN, including NCV testing and bedside neurological examinations such as vibration perception, monofilament testing, and tendon reflex assessment, remain the current clinical standards (40). However, these methods have several drawbacks: NCV requires specialized equipment and trained personnel, is time-consuming, and is poorly suited for large-scale screening, while bedside tests often lack sensitivity for early or subclinical disease and are subject to inter-observer variability (40). In contrast, serum biomarkers such as NfL and Tau can be measured using routine immunoassays, offering an objective, minimally invasive, and reproducible alternative. In our study, although both NfL and Tau were individually associated with DPN, their combined assessment markedly improved diagnostic accuracy, thereby outperforming either biomarker alone and complementing the limitations of traditional diagnostic techniques. The combined assessment of serum NfL and Tau may serve as a complementary tool for screening individuals at risk of diabetic peripheral neuropathy.

Several limitations should be acknowledged. First, this was a retrospective, single-center study with a relatively small sample size, which may limit generalizability. Second, the cross-sectional design precludes conclusions regarding causality or the prognostic value of NfL and Tau for predicting DPN progression. Third, potential confounders such as comorbidities, medication use, and metabolic control were not fully accounted for. Last but not least, because NfL and Tau are not specific to peripheral nerves and may reflect global neuroaxonal injury arising from both central and peripheral nervous system involvement, larger multicenter cohort studies with sufficient sample sizes are required to further validate their utility as screening biomarkers. Future multicenter prospective studies with larger cohorts are needed to validate our findings and to establish standardized cut-off values for clinical use. In addition, mechanistic studies are warranted to clarify the role of Tau in peripheral neuropathy and to evaluate whether combined biomarker strategies can be integrated into current screening pathways for DPN.

The findings of this study have important clinical relevance. Serum biomarkers such as NfL and Tau can be measured using standard immunoassays, offering a minimally invasive, reproducible, and relatively low-cost approach compared with neurophysiological testing. Incorporating NfL and Tau into screening protocols may improve early detection of DPN, facilitate risk stratification, and allow timely initiation of preventive or therapeutic interventions. Moreover, these biomarkers could potentially be used to monitor disease progression and response to treatment in both clinical trials and routine practice.

## 5 Conclusion

In summary, this study demonstrates that serum NfL and Tau proteins were significantly elevated in patients with DPN and show strong associations with impaired nerve conduction. Logistic regression analysis identified both biomarkers as factors independently associated with DPN, while receiver operating characteristic curve analysis indicated that their combined assessment provides superior diagnostic accuracy compared with either marker alone. These findings highlight the potential clinical utility of NfL and Tau as complementary, non-invasive biomarkers for the identification of DPN. Importantly, these findings also support the need for greater preclinical standardization in future translational studies, including the use of well-defined diabetic neuropathy models, standardized timing of biomarker sampling across disease stages, and harmonized analytical platforms for NfL and Tau quantification. Such standardization will facilitate the clinical translation of NfL and Tau as complementary, non-invasive biomarkers for the identification and monitoring of DPN.

## Data Availability

The raw data supporting the conclusions of this article will be made available by the authors, without undue reservation.
